# Bio-Food Quality, Environmental Pollution, and the Role of Algae in Promoting Human Health and Sustainability

**DOI:** 10.3390/life14111513

**Published:** 2024-11-20

**Authors:** Lavinia-Lorena Pruteanu, Roxana Mare, Beatrice Mihalescu, Lorentz Jäntschi

**Affiliations:** 1 Department of Chemistry and Biology, North University Center at Baia Mare, Technical University of Cluj-Napoca, 430122 Baia Mare, Romania; 2 Faculty of Building Services Engineering, Technical University of Cluj-Napoca, 28 Memorandumului Street, 400114 Cluj-Napoca, Romania; 3 Department of Physics and Chemistry, Technical University of Cluj-Napoca, 400114 Cluj-Napoca, Romania; lorentz.jantschi@gmail.com

**Keywords:** pollution reduction, environment, soil composition, solar radiation, algae, health, food, quality of life

## Abstract

Healthcare resources have changed fundamentally compared to decades ago. Modern bio-food products and sustainable solutions for their production have increased the attention of researchers, taking into account the current level of pollution of the earth and atmosphere along with modern technologies applied to processed foods. Therefore, this review aims to highlight: (1) the impact and relationship between the physiological parameters of the atmosphere, solar radiation and soil, (in terms of their composition and stages of formation and organization) along with the evolution to modern life; (2) the environmental impacts on algae, living organisms, food, and human health and sustainability. In addition, we address the significant impact of algae as a sustainable resource in reducing environmental pollution contributing to a healthier life.

## 1. Introduction

Human health depends on quality of life, which is directly influenced by the quality of the environment and food. Unfortunately, the quality of the environment and food is strongly affected by (i) the increasing emissions of potentially toxic gases and substances into the atmosphere and soil [1], which come with technological progress in recent years, and (ii) industrial sources of hazardous chemicals (i.e., heavy metals, plastics, and pesticides) [2,3].

The alarming evidence is generally related to foods, and in addition nutrients. In algae, the nutrient composition changes; they can accumulate different pollutants together with nutrients [4]—micro-plastic is one of the most dangerous because it shows interactions during algal blooms [5]. The result is water with low oxygen levels, which is very dangerous for living marine organisms (fish, crustaceans, etc.) [6,7,8]. All these negative changes in organisms are further reflected in the degradation of the environment, food, and human health—aspects reflected by the increased number of diseases among populations [9,10].

Worrying reports of high pollution levels have increased scientists’ interest in exploring and developing green technologies such as gas hydrate combustion technology used to burn methane gas hydrate reduces harmful emissions [11]; green technologies [12,13] that promote the minimization of environmental damage by reducing carbon dioxide emissions (CO_2_E) [13]; the integration of microalgae in wastewater treatment [14]; or other sustainability strategies to reduce environmental pollution [15] such as algal clothing [16], algal biofuels as sources of microalgae (phytoplankton) and oils containing macroalgae [17,18,19], biodegradable bottles with algae [20,21] etc.

Despite the recent trend of mitigating environmental damage that has occurred over the years, it is possible that irreversible damage has already occurred, and humanity has concluded it is currently operating outside the planetary boundary [22]. Therefore, this leads to sustainable solutions.

Environmental pollution that affects organisms and ultimately human health, induces oxidative stress at the cellular level; this stress can be reduced or eliminated by the antioxidants found in algae. Also, the need to cover the lack of minerals that the body used to consume naturally led researchers to promote higher bio-accessible fractions of mineral elements in functional foods by fortifying common food products (such as bread) [23,24]. In addition, researchers have expanded the field of using algae for better life and health and have shown that certain algae extracts have therapeutic effects and a high potential for use in the treatment of various diseases (such as cancer). For example, fucoxanthin, a xanthophyll from brown algae was shown to inhibit the PI3K pathway alone and in combinations enhancing the activity of other known PI3K inhibitors in glioblastoma [25].

Another clear evidence of the impact of technology on the environment and influencing its pollution levels was recently demonstrated during the pandemic restrictions; during that time, the environment tended to reach healthier parameters. This was demonstrated in biologically activated sludge from wastewater treatment plants [26], and in surface and groundwater [27] where heavy metals concentrations were reduced or removed by using different species of algae [28,29,30].

Considering all the presented aspects, this review aims to highlight: (1) the impact and relationship between the physiological parameters of the atmosphere, solar radiation, and soil, (in terms of their composition and stages of formation and organization) together with evolution to modern life; (2) environmental impacts on algae, living organisms, food, and human health and sustainability. Its significance lies in addressing the potential of algae for environmental detoxification, providing insights into how algae can be both a nutrient resource and an environmental solution. Basically, this review examines the intersection of bio-food quality, environmental pollution and the role of algae in promoting human health and sustainability.

## 2. Understanding the Role of the Earth and the Structure of the Atmosphere and Their Impact on Quality of Life

Understanding the chemical composition of the atmosphere allows us to understand variations that may pose potential risks to the quality of life on Earth. Atmospheric pollution is the result of human activities [31] that determine the deterioration of the quality of the atmosphere. Damage reduction can be achieved by limiting the presence of risk factor contaminants in the atmosphere.

**Figure 1:**
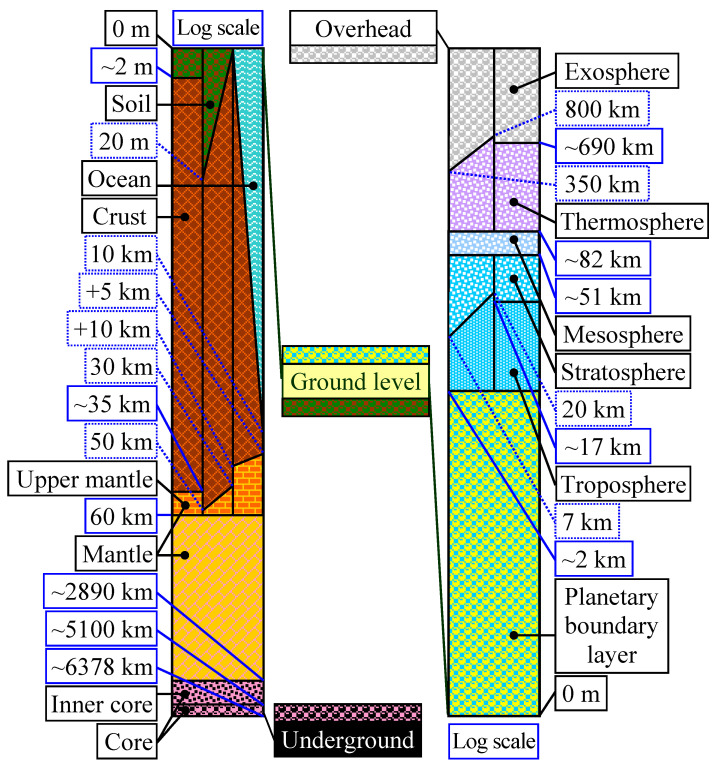
Logarithmic scale representation of the structure of the Earth and atmosphere: p. 34 in [40].

The logarithmic scale in Figure 1 emphasizes the complex structure of the Earth and atmosphere, showing first the levels that are in direct contact with biological matter (planetary boundary layer, soil, and natural waters), and ending with the outermost levels (exosphere and center of the Earth) and/or with algae.

Most algae species are aquatic, which is why the distinct types of cells and tissues (i.e., stomata, xylem, and phloem) found in terrestrial plants are lacking. However, modern applications of algal culture: (i) go beyond food traditions and include cattle feed [32,33]; (ii) use algae for bioremediation or pollution control [34,35]; (iii) convert sunlight into algae fuels [36,37] or other chemicals used in industrial processes; and (iv) medical and scientific applications [37,38,39].

Fertile soil is on average up to 2 m in the subsoil (Figure 1) with a thickness varying from 0 (in desert areas) up to 20 m (in very rich vegetation areas) with Af zones in the Köppen–Geiger climate classification [41] and intensively interacts with biological organisms, causing it to have a special composition. Alternatively, the same area of depth may be covered by water (such as running waters, seas, and oceans). In the case of oceans, the depth can vary up to 10 km. The next area with a characteristic composition is the Crust; its thickness can vary from 5 km (in the case of the oceans) to 30 km (in the case of land covered with vegetation) to 10 km (in the case of the oceans) and 50 km, respectively (in the case of land covered by vegetation) with an average thickness of 35 km in the latter case.

The crust continues through the upper mantle and reaches a depth of about 60 km. The mantle is the largest surface (approx. 81%) and extends to approx. 2890 km, followed by the inner core (approx. 16%) up to 5100 km, and the core (by less than 1%, up to approx. 6378 km). The underground interacts intensely with biological organisms (the planetary boundary layer-up to 2 km high), causing a significantly different composition. Compared to level 0 (ground level), the atmosphere is located above. Next, the troposphere (7–20 km, with an average value of 17 km), continues with the stratosphere (up to 51 km), the mesosphere (up to 82 km), the thermosphere (from 350 km to 800 km with an average value of 690 km) and the exosphere.

Regardless of the type of the ecosystem (be it aerial, terrestrial, or aquatic), they are all affected by pollution (i.e., exhaust emissions from cars are purged into the atmosphere, while exhaust gas emissions from the planes are purged into the atmosphere and troposphere).

**Figure 2:**
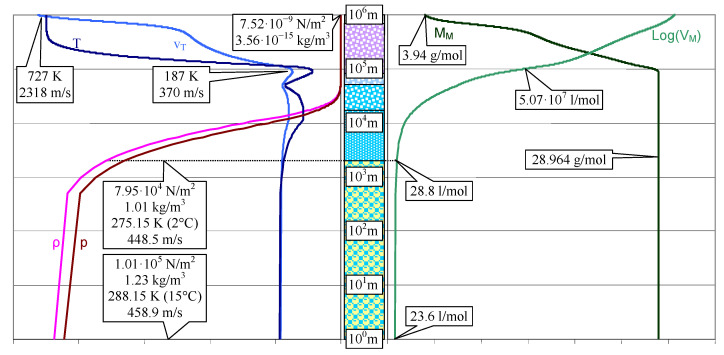
Representation of the physico-chemical parameters of the atmosphere on a logarithmic scale: p. 36 in [40].

Using data from ASTM G173-03 [42], the atmosphere’s physicochemical parameters can also be represented on the logarithmic scale (Figure 2).

The most informative property regarding the atmosphere’s composition is the molar mass. It practically does not vary up to 100 km; its average value is 28.964 g/mol, an intermediate value between 28.8 (corresponding to an O_2_:N_2_ ratio of 1:4) and 29.0 (corresponding to an O_2_:N_2_ ratio of 1:3). These values precisely reflect the composition of the atmosphere (for 22% oxygen and 77% nitrogen, the average value of the molar mass is 28.89 g/mol). Above 100 km, the molar mass has an approximately linear progression towards 3.94 g/mol in a double logarithmic scale (on both axes); this is an informative value, providing, again, with great precision, the composition of the extra-atmospheric space (mixture of Helium and Hydrogen).

The logarithmic scale representation of the atmosphere’s physicochemical parameters shows the correlated relationship between pressure *p* and density *ρ* on one hand, and between temperature *T* and thermal velocity *v_T_*, on the other. It is expected to have these associations if it approximates an ideal gas for which *R* is the gas constant, *M* represents the molar mass, and *J* is the number of velocity components. These parameters are linearly (*pM* = *ρRT*) and monotonically (*v_T_*^2^*M* = *JRT*) associated. Moreover, pressure and density significantly influence the absorption of solar radiation which can considerably influence the existence of living organisms (including the algae), food quality, and human health.

**Figure 3:**
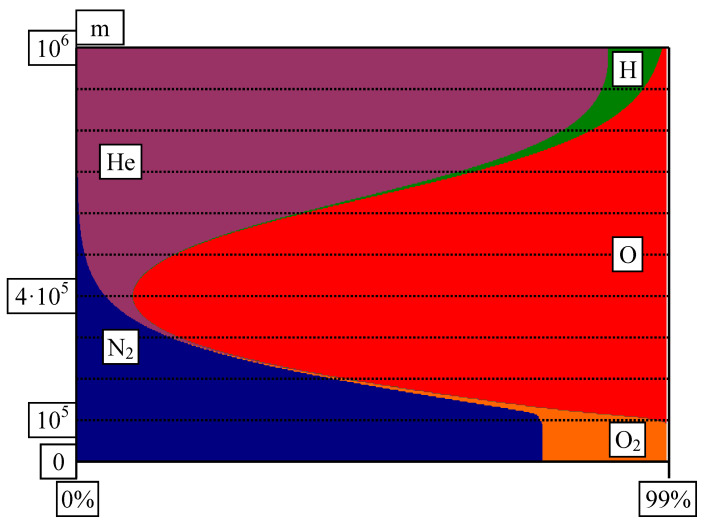
The extra-atmospheric space composition (100 km to 1000 km): p. 40 in [40].

The life and survival of living organisms are dependent on the atmosphere’s composition (as shown in Figure 3). In total, 99% of the atmosphere consists of N_2_, O_2_, O, He, and H. Up to 100 km, the atmosphere consists of molecular oxygen and nitrogen, essential for the existence of living organisms. From this point up to 1000 km, molecular oxygen dissociates into atomic oxygen. First, molecular oxygen is replaced by atomic oxygen. Further, atomic oxygen replaces molecular nitrogen, too, due to its stability (triple bond in N_2_) and weight (M(N_2_) = 28 g/mol, M(O) = 16 g/mol). Atomic oxygen is necessary for the equilibrium between ozone and oxygen to occur (O_3_ ↔ O + O_2_). Ozone absorbs most of the long-wavelength solar radiation (UltraViolet UV-B and UV-C, 100–315 nm), protecting the biological matter from its germicidal effects. Starting at 400 km, the abundance of atomic oxygen begins to decrease in favor of helium; atomic hydrogen also begins to appear in a small fraction.

## 3. The Impact of the Atmosphere on the Effects of Solar Radiation

**Figure 4:**
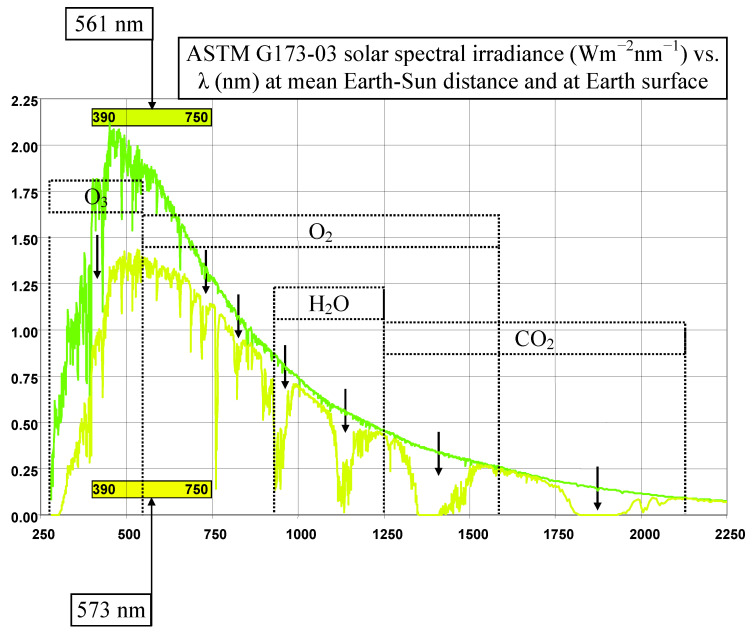
Dependence of the solar intensity on the wavelength at the entrance to the atmosphere (at 1000 km height): p. 34 in [40].

About 70% of the radiation received from the Sun is absorbed by the atmosphere (27%) and the Earth’s surface (43%); the remaining part is reflected into space and does not heat the surface [43]. The intensity of solar radiation depends on the wavelength at the entrance to the atmosphere (at 1000 km height) and the ground surface (according to data from ASTM G173-03) (Figure 4).

As shown in Figure 4, the atmosphere absorbs more radiation with shorter wavelengths than radiation with longer wavelengths. The ultraviolet (UV) radiation together with the visible range are the most absorbed in the atmosphere by O_3_, O_2_, H_2_O, and CO_2_ (in order of increasing wavelength absorption). Most UV radiation is absorbed by the ozone layer; however, UV waves reach the Earth’s surface and water, also affecting living organisms and algal systems to a lesser or greater extent (e.g., they can affect growth, photosynthesis, nitrogen incorporation, and enzyme activity) [44]. 

Moreover, the average value of the solar intensity in the visible range changes from 561 nm at the entry into the atmosphere (corresponding to a yellow-green color, but what is seen is the complementary color, from yellow to red) to 573 nm (corresponding to an intense yellow color.

## 4. Ozone Layer

The ozone layer or shield is a region of the Earth’s stratosphere, from 7 to 51 km (see Figure 1). It contains a high concentration of ozone (O_3_) compared to other parts of the atmosphere but is still lower compared to other gases in the stratosphere (which contains N_2_, O_2_ and O_3_, and reactive nitrogen species) [45]. The ozone layer is essential for absorbing most of the Sun’s ultraviolet radiation. Although the concentration in the ozone layer is very low, it is vital to life because it absorbs biologically harmful ultraviolet (UV) radiation from the Sun. Ultrashort or vacuum UV (10–100 nm) is shielded by N_2_. The rest of the UV radiation (100 nm to 400 nm) is divided into three categories: UV-A (400–315 nm), UV-B (315–280 nm), and UV-C (280–100 nm). UV-C, which is very harmful to all living organisms, is completely removed by a combination of O_2_ (<200 nm) and O_3_ (>about 200 nm) in the stratosphere ($O_{2}\;\overset{h\nu \;\mathrm{from}\;\mathrm{UV}}{\to }2O$, $O+O_{2}⇌O_{3}$). UV-B radiation can be harmful to the skin, being the main cause of sunburn; excessive exposure can also cause cataracts, immune system suppression, and genetic damage, leading to problems such as skin cancer.

The O_3_ layer absorbs radiation with wavelengths from about 200 nm to 310 nm [46,47]; it is effective at removing UV-B radiation (for example, radiation with a wavelength of 290 nm has an intensity at the top of the atmosphere 350 million times stronger than at the Earth’s surface). Some of the longest-wavelength UV-B radiation reaches the Earth’s surface, which is important for the skin’s synthesis of vitamin D. Ozone absorbs very little UV-A, accounting for most of the UV reaching the Earth. Even though this UV radiation is less damaging to DNA, it can still cause physical damage, premature skin aging, indirect genetic damage, and skin cancer [48,49,50,51].

## 5. The Influence of Soil Components on Quality of Living, Organisms and Food

**Figure 5:**
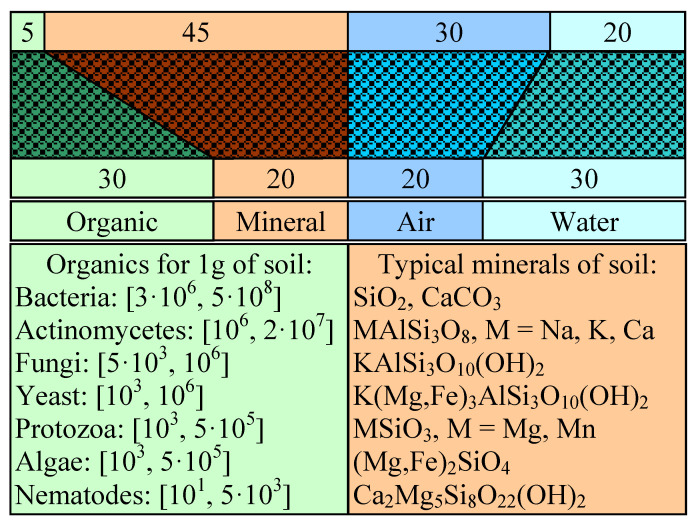
Chemical compound in soil: p. 39 in [40].

About half of soil is fluid (liquids or gases) and the other half is originally half organic and half inorganic. Silicon dioxide is the most abundant chemical compound contained in various forms of silicates, aluminosilicates, and hydroxy-aluminosilicates. The abundance of living organisms in fertile soil is considerable (Figure 5). But this abundance is strongly influenced by the thickness of the fertile soil layer—if the land is covered with vegetation (forests, meadows) then it has a considerable thickness; if the terrain is modified, (stone or asphalt), its thickness is 0 or close to 0. Fertile soil condition is essential for plant growth and oxygen production.

**Figure 6:**
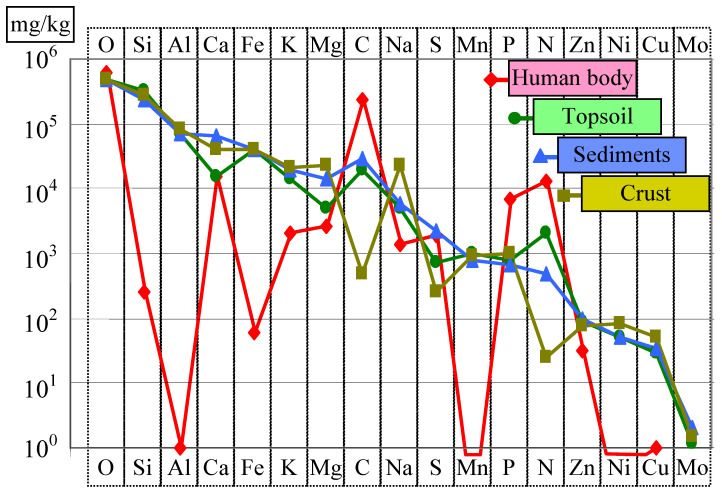
The chemical composition of various systems containing organic matter: p. 40 in [40].

Living organisms in various systems containing organic matter concentrate certain chemical elements at the expense of others. We discuss the land surface, sediments, and crust, in comparison with the human organism which is considered as a reference (Figure 6). Aluminum, present in large proportions in soil, together with manganese, is filtered and removed by living organisms (see the red line in Figure 6). Instead, organic elements (C, N, P, etc.) are concentrated. Interestingly, trace elements (i.e., Fe, Mg, and Ca) are also less represented in living organisms than in the environment located in their immediate vicinity.

**Figure 7:**
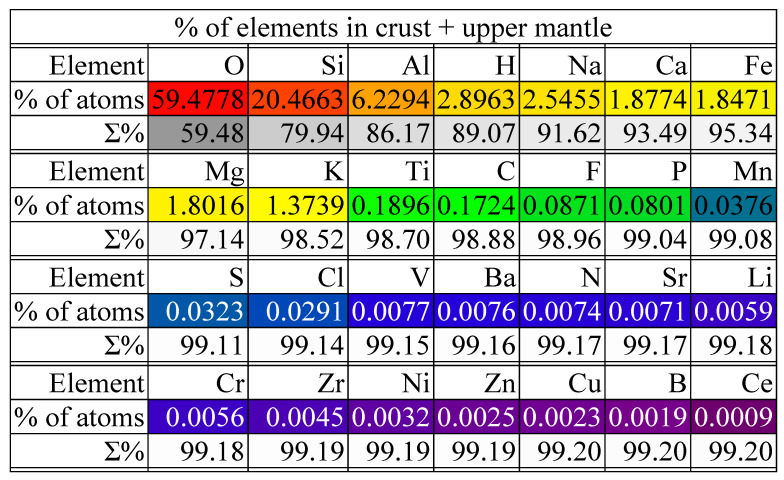
The chemical composition of the crust and upper mantle (with Σ% means the cumulative expressed as a percentage): p. 41 in [40].

Moving on to the crust and upper mantle, Figure 7 illustrates a rather little-known fact: oxygen is the most abundant (about 59% of the atoms are oxygen atoms—which is the largest proportion) compared to air, which has only about 20% oxygen atoms, and about 33% water. The crust and upper mantle are rich sources of oxygen. Therefore, the main minerals in the two layers are hydroxy-aluminosilicates (MAlSi_3_O_10_(OH)_2_) (Figure 5), the chemical formula that explains the appearance of the first four elements in order of abundance (Figure 7).

**Figure 8:**
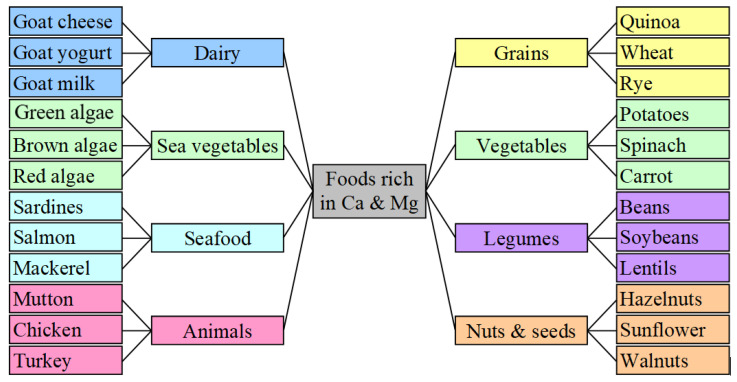
Calcium and magnesium resources: p. 162 in [40].

Among the divalent metal cations, Calcium and Magnesium are the most abundant. The figure below illustrates their main dietary resources (Figure 8).

**Figure 9:**
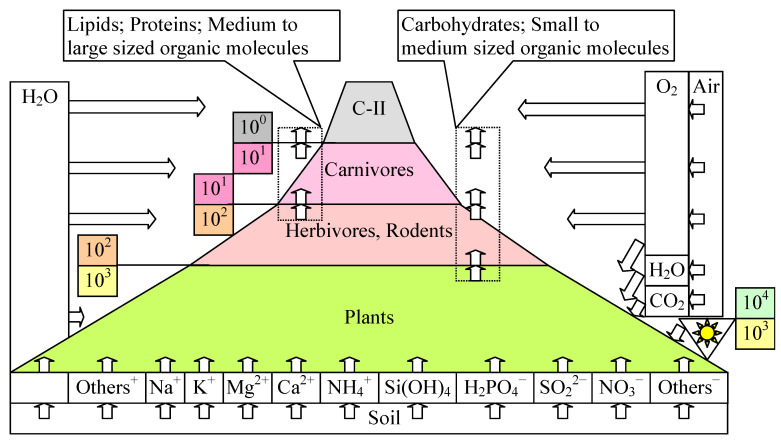
Trophic chain: p. 182 in [40].

Plants interact with the soil (from which they extract water and minerals) and the atmosphere (from which they extract CO_2_ and H_2_O in the presence of light and O_2_ in its absence) (Figure 9) to produce biomass corresponding to a solar energy conversion efficiency of about 10%. In the trophic chain, this biomass is used by herbivores and rodents to further produce a total mass of approx. In total, 10% of the total mass of plants is preyed upon by carnivores, whose total mass is also approx. 10% of the total mass of herbivores and rodents. Humans also play an important role, as their total mass is again about 10% of the total mass of carnivores. Therefore, this proportion of 10% is very important, because it gives a balance to the ecosystem according to the Hardy–Weinberg principle [52,53].

Figure 9 shows the preference of plants for certain ions by which they secure their minerals (Na^+^, K^+^, Mg^2+^, Ca^2+^) and organoelements (nitrogen, sulfur, and phosphorus). Carbon, oxygen, and hydrogen are the only ones that come from the atmosphere. For the rest of the organisms, the carbon requirement is also ensured by the interaction with the lower levels in the trophic pyramid. On this food chain, the complexity of the synthesized molecules (lipids, proteins, carbohydrates) also increases.

**Figure 10:**
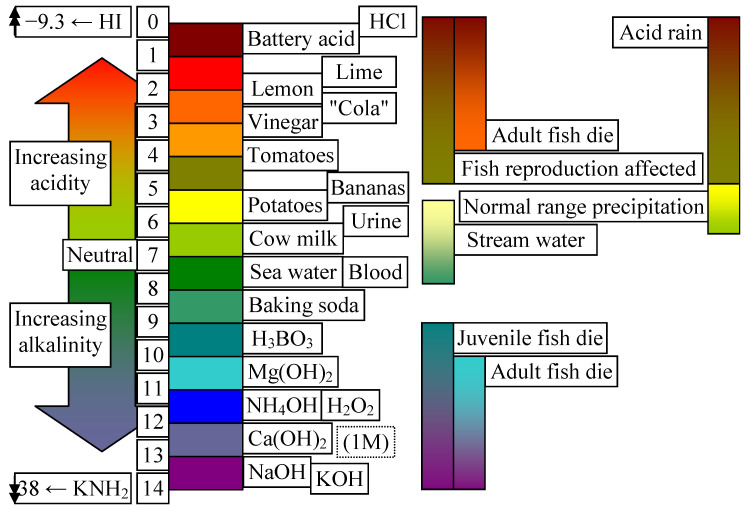
Different representative values for pH and their influence on living organisms: p. 128 in [40].

Another important factor for living organisms is pH. With this in mind, the plot in Figure 10 illustrates the variation in pH and its influence on various living organisms.

Marine algae strains prefer a pH typically around 8.1 (with a tolerance of 5.0 to 9.7) [54,55], while freshwater strains are less adapted, preferring a pH around 7.0 (with an average of 6.3 to 9.3) [56,57]. Generally, a pH value between 7.0 and 9.0 supports algae growth.

**Figure 11:**
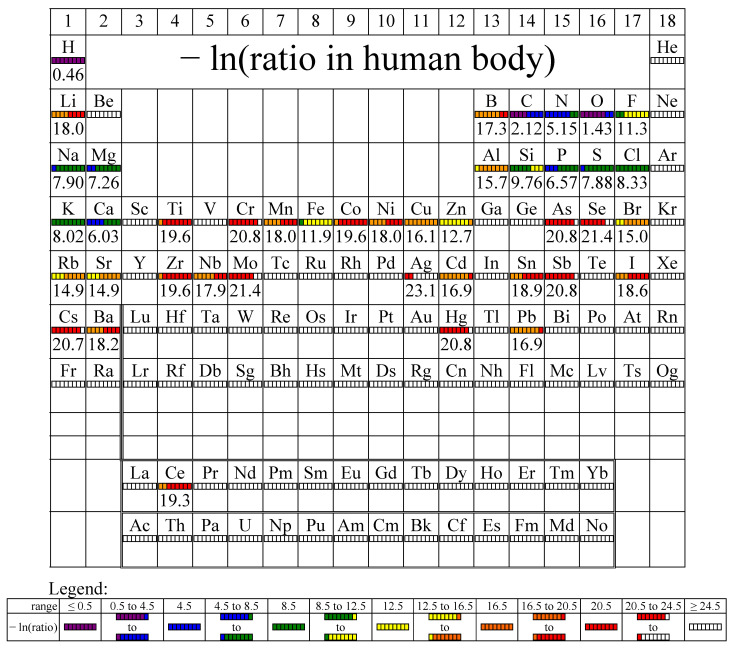
Map of the chemical elements represented in the human body (adapted from p. 40 in [40]).

Figure 10 is an important illustration of the dissociation of the effect of water on living biological systems, while Figure 11 shows a map representation of the chemical elements in the human body, with values on a logarithmic scale representing the fraction.

Thus, the acceptable range of water dissociation and pH is from 4.0 to 9.5 on pH scale; pH values outside this range are harmful to most living organisms. The extreme limits of pH (about 0 for HCl and about 14 for KOH) are exceeded by several substances that have a strong dissociating effect on water (i.e., each molecule of KNH_2_ with one molecule of water forms two bases: KOH and NH_3_, which consumes the amount of undissociated water and artificially increases the pH value).

## 6. Reducing Environmental Pollution and the Role of Algae on Quality of Life and Human Health

**Figure 12:**
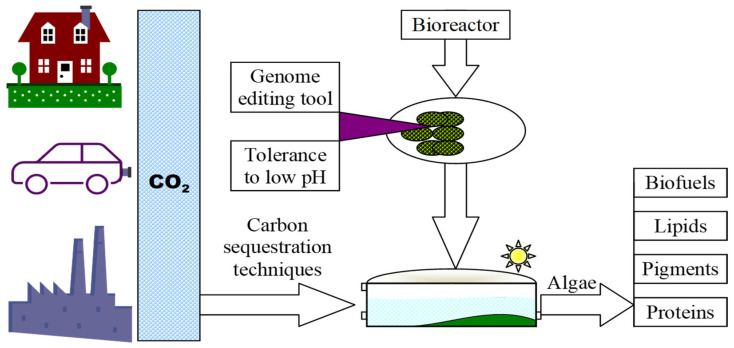
Schematic representation of CO_2_ captured from the environment and its transformation into sustainable biomaterial (adapted from p. 409 in [40] and [58]).

Algae are plant-like organisms that photosynthesize and are found in the sea, land, and fresh water. Microalgae can be prokaryotes (cyanobacteria) or eukaryotes (green algae), which can fix 10–15 times more CO_2_ than other terrestrial plants. They multiply rapidly and have a high potential to fix carbon from the atmosphere (Figure 12) and convert it into bioenergy, making it a sustainable biomaterial to produce many high-value products [58,59]. This biological method of capturing and transforming carbon from the environment is more effective than physical methods of ameliorating environmental pollution [60,61].

**Figure 13:**
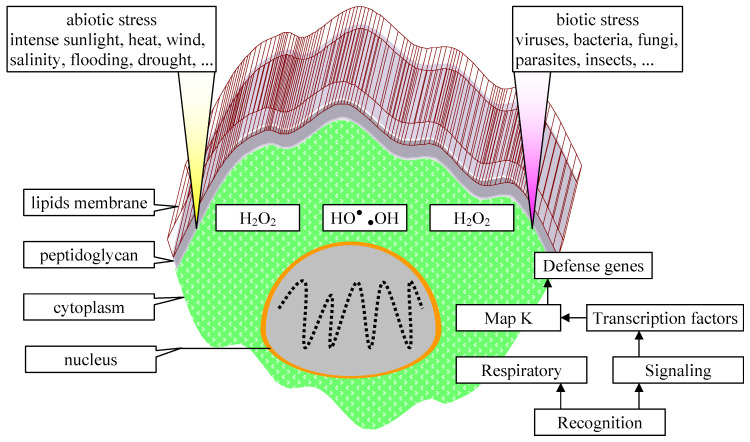
Cellular stress and external stress factors (adapted from [64] and p. 22 in [65]).

Phytoremediation, or the use of microalgae to mitigate organic and inorganic contamination, offers advantages such as the remediation of industrial and domestic waters and those contaminated with heavy metals [62]. Microalgae cells can accumulate heavy metals up to 10% of biomass due to their high surface-to-volume ratio, with efficient metal binding, uptake, metabolism and storage mechanisms [63]. Changes occur at the cellular level once environmental changes occur. There is a direct link between cellular stress and external stress factors such as pollution (Figure 13), which affect defense gene mechanisms. Once the stress factor is recognized, the signals affect the activity of the transcription factors and implicitly the pathways in which the genes are involved.

In agriculture, the use of chemical fertilizers has generated environmental pollution and loss of soil fertility; this has resulted in poor food quality and human health. Because of this and because of the food composition of the world based mainly on vegetables (especially traditional grains: wheat, rice, corn, barley, etc.) and meat (beef, poultry, and pork) [66], concerns about the environmental impact of existing food production systems together with health problems have created the need to develop new, more sustainable, and healthier food sources [67].

This is where the microalgae and bacteria identified as alternatives for improving soil fertility come into play. It is due to biofertilizing properties through the production of phytohormones, amino acids, carotenoids and their ability to inhibit plant pathogens [68]. Lichner et al. performed an experimental inoculation of microalgae/cyanobacteria in sandy soil; the aim was to improve the concentration of soil nutrients such as nitrogen, phosphorus, organic carbon and other minerals. Soil stability, soil water infiltration and moisture content were considered. They considered the hydrophysical properties of sandy soil that was or was not treated with algae and found substantial differences between the two soil surfaces [69].

Microalgae, especially cyanobacteria, can also fix atmospheric nitrogen, helping to improve soil nitrogen content. They have special mechanisms for fixing nitrogen from the atmosphere, as they use a complex of nitrogenase enzymes to convert atmospheric nitrogen into ammonia [70]. As a nitrogen source, microalgae are applied to the soil as a live culture in the case of cyanobacteria or as dry biomass or suspension in the case of green algae [71].

The food industry is trying to replace synthetic dyes with natural pigments for their coloring ability and healthy properties. Microalgae have proven to be one of the main suppliers of valuable natural pigments in the global food pigments market. *Chlorophylls*, carotenoids, and phycobiliproteins are pigments derived from microalgae, which have unique colors and molecular structures, and exhibit various physiological activities with effects on human health [72].

Several bioactive compounds have been discovered and purified from marine microalgae, such as sulfated polysaccharides, and various carotenoids (fucoxanthin, β-carotene, astaxanthin, omega fatty acids, polyphenols). Some of these metabolites have demonstrated strong antioxidant, anti-inflammatory, anti-cancer, and antiviral properties [73]. They have great potential as supplements in the human diet for the prevention and treatment of physiological conditions instead of synthetic food supplements [74].

## 7. Aquatic Pollution Reduction and the Role of Algae on Human Quality of Life and Health

Pollution has significant negative effects on the aquatic environment regarding:

- Water Quality: Pollution from sources such as industrial waste, agricultural runoff, and sewage can degrade water quality, making it unsafe for aquatic organisms and even humans.
- Habitat Destruction: Pollution can destroy or disrupt aquatic habitats through factors such as chemical contamination, sedimentation, and eutrophication (excessive nutrient enrichment).
- Loss of Biodiversity: Pollutants can harm or kill aquatic plants and animals directly or indirectly by disrupting food chains and ecosystems.
- Oxygen Depletion: Certain pollutants contribute to the depletion of oxygen in water bodies, leading to hypoxic (low oxygen) conditions that threaten aquatic life.
- Bioaccumulation: Pollutants such as heavy metals or persistent organic pollutants can accumulate in the tissues of organisms over time, posing health risks to both wildlife and humans who consume contaminated fish or shellfish.
- Altered Behavior and Reproduction: Exposure to pollutants can affect the behavior, reproduction, growth, and development of aquatic species.

Efforts to address pollution in the aquatic environment involve the implementation of regulations regarding discharges into water bodies; promoting sustainable practices in industries, agriculture, and urban areas; improving wastewater treatment technologies; water quality monitoring; carrying out environmental impact assessments; and increasing public awareness of the harmful effects of pollution on ecosystems.

Unfortunately, in order to protect the public during the COVID-19 pandemic, medical masks were used, and through the improper management of the waste from them, an increase in marine pollution in terms of water quality, but also a decrease in aquatic microorganisms. Waste plastics, such as polymer-based disposable surgical masks, contain various types of chemical additives, stabilizers, plasticizers, bisphenol A, and phthalate [75], which affect the ability of the material to degrade [76]. The chemical compounds released from their decomposition have a negative impact on the microorganisms in the aquatic environment [77]. Researchers such as Hazeem et al. analyzed their harmful effects on the microalgae *Chlorella vulgaris (Chlorophyta)*, and the results showed cell damage, a decrease in the content of proteins, lipids, nucleic acids, and polysaccharides, resulting in a negative impact on their development, which led to a decrease in the quality of their biomass [78].

Other studies show the effect of micro- and nano-plastics on the photosynthetic activity of aquatic photoautotrophs, especially on microalgae and cyanobacteria [38,75]. To study this impact, pigment content and photosynthesis rate were used as indicators. Wang et al. found that polyvinyl chloride inhibited the chlorophyll content of algae, and the effect was directly proportional to the concentration [79]. Chen et al. showed that polystyrene reduced the content of chlorophyll a, chlorophyll c, and carotenoids in cells of *Phaeodactylum tricornutum (Bacillariophyceae)* [80]. Some studies have shown that exposure to other types of micro- and nano-plastics does not have a significant impact, it may even stimulate the photosynthesis of aquatic photoautotrophs [81]. For example, amino-modified polystyrene (-NH_2_) does not affect photosynthesis in *Chaetoceros neogracilis (Mediophyceae)* [82], nor does carboxylated polystyrene in *Dunaliella tertiolecta (Chlorophyta)*.

In certain aquatic ecosystems, algae act as primary producers, synthesizing organic material and oxygen for the metabolism of consumer organisms. As species that live in extreme environments, with variations in some factors, they need readaptation to the changes that occur, producing primary metabolites (proteins, amino acids, polysaccharides, and fatty acids) that act in response to changes in the environment [83].

## 8. Conclusions

This review provides a wide range of information on some basic features of the Earth’s environment at very large scales, including the atmosphere, Earth’s surface structure, and life systems in terms of physical and chemical attributes.

This context is used to more directly address the possible role of algae in ameliorating some of the increasing pollutant effects and/or increasing the productivity and salience of our natural environment, including improving agricultural productivity and the quality of human life.

Therefore, this paper incorporates the intersection of bio-food quality, environmental pollution, and the role of algae in promoting human health and sustainability with its significance, which lies in addressing the potential of algae for environmental detoxification, providing insights into how algae can be both a nutrient resource and an environmental solution.

We can only conclude and emphasize that environmental factors (such as atmospheric conditions, solar radiation, and soil composition) affect the quality and bioactive potential of bio-food products, especially algae. Algae and a balance in their ecosystem are very beneficial to the environment, preventing other hazards, unhealthy life and improving quality of life.
